# Preparation and characterization of high-performance ceramic proppant from recycling utilization of oil-based drilling cuttings pyrolysis residues

**DOI:** 10.1038/s41598-024-52334-7

**Published:** 2024-01-29

**Authors:** Yuanyi Yang, Hui Li, Zhenghuan Lei, Hongwu Liu, Mingyou Zeng, Tingting Yang, Keming Chen, Yi Duan

**Affiliations:** 1 School of Civil Engineering and Geometrics, Southwest Petroleum University, Chengdu, 610500 China; 2 CNPCCCDE, Safety Environment Quality Surveillance & Inspection Research Institute, Guanghan, 618000 China; 3 College of Environmental Science and Engineering, China West Normal University, Nanchong, 637009 China; 4 Research Institute of Engineering Safety Assessment and Protection of Southwest Petroleum University, Chengdu, 610500 China

**Keywords:** Pollution remediation, Pollution remediation, Solid-state chemistry, Ceramics

## Abstract

Oil-based drilling cutting pyrolysis residues (ODCPRs), bauxite, and sintering additives were applied to manufacture ceramic proppants with low density and high strength in this work. The effect of ODCPRs ratio, sintering temperature, holding time, and the content of additives on the performance of the proppants was comprehensively investigated, respectively. And the sintering mechanism of proppants was also discussed according to the phase, microstructure, and thermal behavior analyses. The results revealed that at the best sintering condition (1280 °C, holding for 60 min), and a mass ratio (ODCPRs: bauxite: MnO_2_ at 3:7:0.1), the well-developed granular corundum and acicular mullite formed inside the proppants and interspersed with each other to form a dense structure. The proppants presented low density and high strength as the bulk density of 1.48 g/cm^3^, the apparent density of 2.94 g/cm^3^, a breakage ratio of 5.25% under 52 MPa closed pressure, and the acid solubility of 4.80%, which could well meet the requirement of the standards of SY/T 5108-2014. This work provided a new pathway for recycling ODCPRs and the fabrication of high-performance proppants.

## Introduction

With the rapid economic development of China, new energy source exploitation and application have become the most critical factor in solving the problem of the huge energy demand of the entire society^1,2^. Shale gas is an important new energy source with extremely large reserves in China^3^. However, with the fast growth of the shale gas industry, a mass of hazardous solid wastes such as oil-based drilling cuttings (OBDC) have been generated during the drilling process^4^. Due to the rather complex compositions of the OBDC, which mainly contains heavy metals, organic compounds, petroleum hydrocarbons, and soluble salts, the direct discharge of OBDC will cause serious environmental and human health damage^5^. Thus, it is a hot topic in studying the efficient treatment and further recycling utilization of OBDC.

Currently, pyrolysis technology is the most commonly used harmless industrial treatment of OBDC^6–8^. However, after the pyrolysis process, the primary characteristic pollutants in oil-based drilling cuttings, such as petroleum hydrocarbons and heavy metals, still exist in the pyrolysis residues^9^. The random discharge or landfilling of the oil-based drilling cutting pyrolysis residues (ODCPRs) will still occupy lots of land resources and even cause potential pollution risks^10^. Therefore, it is necessary to develop green, safe, and environmentally friendly resource recycling utilization approaches for ODCPRs. Researchers have extensively explored the resource utilization of ODCPRs to prepare building materials, for example non-fired^11^ or fired bricks^12^, hot mix asphalt^13^, cement clinker^14,15^, oil well cement^16^, concrete aggregate^17,18^, subgrade materials^4^, glass–ceramic materials^19^. Nevertheless, the manufacture of these building materials from ODCPRs is costly and energy-intensive, while the resource utilization products are usually low value-added. Even more importantly, the ODCPRs recycling capacity for building materials preparation is still very limited, which cannot completely consume such a mass of ODCPRs waste^20^. Thus, it is necessary to explore new feasible resource utilization methods.

Hydraulic fracturing technology is widely adopted to increase the exploitation of oil and gas resources output^21,22^. Proppants are the key materials in the hydraulic fracturing process to remain the cracks opening after the hydraulic pressure is removed^23^. Ceramic proppants perform better than quartz sands due to their high mechanical strength and good chemical stability. But the ceramic proppant costs more and has a higher density resulting from the raw materials of high-alumina bauxite, and higher fracturing operation conditions are also required. Therefore, exploring low-cost, low-density, and high-breakage-resistance ceramic proppants is urgent. Alternative materials and extra additions have been used to fabricate ceramic proppants, including kinds of industrial solid wastes, such as fly ash^23^, lower-grade bauxite^24^, spent Cu-based oxygen carriers^25^, and water-based drill cuttings^20^, which has confirmed the feasibility of waste resource recycling for proppant preparation. Simultaneously, the chemical compositions of ODCPRs also contain some rewarding components, for instance: calcium oxide, barium oxide, magnesium oxide, and iron oxide, which are proven to be valuable for reducing the sintering temperature^26,27^, density^28,29^, breakage rate^28,30^, and acid solubility^30,31^, etc. Thus, ODCPRs can also be used to prepare ceramic proppants with other mineral materials, not only decreasing the ODCPRs accumulation but also reducing the production cost of proppants.

This work aims to recycle ODCPRs as a raw material to prepare high-performance ceramic proppants and to identify the optimal preparation processes through the property evolution of ceramic proppants under different mix designs and sintering systems. The performance of the proppants with different sintering temperatures, holding times, and various ratios of ODCPRs to bauxite are comprehensively investigated. Besides, the effect of V_2_O_5_ and MnO_2_ additives on the proppants` sintering behaviors and properties are also discussed. In addition, through combining phase change, microstructure analyses, and thermal behavior analysis, the formulation and evolution mechanism of lightweight proppants are revealed, providing a new pathway for recycling ODCPRs and fabricating high-performance proppants.

## Experimental

### Raw materials

The ODCPRs were provided by CNPC from a shale gas field in the southwest of China, and the bauxite powder was purchased from Gongyi Xingsong Mineral Products Co. LTD. The chemical compositions of ODCPRs and bauxite were analyzed by X-ray fluorescence spectrum (XRF), as shown in Table S1. The content of SiO_2_ in ODCPRs is significantly higher than that of Al_2_O_3_, and the content of impurities, including Fe_2_O_3_, BaO, CaO, MgO, etc., is particularly higher than in bauxite. This has a considerable impact on the sintering process of ceramic proppants. In addition, the high-grade bauxite was input to improve the molar ratio (Al_2_O_3_/SiO_2_) of the formula, which was crucial for the generation of crystals in ceramic proppants. The phase spectra of ODCPRs and bauxite were analyzed by X-ray diffraction (XRD, PANalytical B.V. Netherlands), and the results were presented in Fig. S1. It can be seen from the results that the main phases of ODCPRs included quartz, barite, limestone, and dolomite, and the main phases of bauxite were corundum and mullite. Besides, V_2_O_5_ and MnO_2_ additives were obtained from Chengdu Kelong Chemical Co., Ltd, China.

### Preparation of ceramic proppants with ODCPRs

The preparation procedure of ceramic proppants was depicted in Fig. S2. Firstly, the ODCPRs, bauxite, and other additives were blended with each other according to various formulas in Table 1. Firstly, the ODCPRs, bauxite, and other additives were blended with each other according to the formulas in Table 1, and a horizontal ball mill was used to grind the raw materials in the wetting condition at a ratio of mixture: water:ball = 1:1:8 for 3 h. The dried paste was crushed and sieved through a 0.25 mm screen to obtain the required mix powders. The particle size distribution of fine powders was tested by a laser particle sizer (MASTERSIZER 2000, Malvern Instruments) as shown in Fig. S3a, and most of the powder size was below 10 μm after the grinding process. Secondly, the powder mixtures were transported to the intensive pelletizer (R02, Jianhu Shenjiang Machinery Co. LTD) for granulation. Subsequently, the fresh pellets were totally dried again and then sintered in a muffle furnace under the sintering system as displayed in Fig. S3b. Finally, the cooled proppant was sieved again through a 20–40 mesh sieve, and the properties of sintered proppants should meet the requirements of SY/T 5108-2014 standard.

**Table 1:** The formula for the preparation of proppant samples.

Sample	Content of raw material (g)						
	ODCPRs	Bauxite	Manganese	Vanadium pentoxide	K	Molar ratio (Al_2_O_3_/SiO_2_)	ODCPRs/(ODCPRs + Bauxite) ratio
A0	100	400	/	/	10.87	3.26	20%
B0	150	350	/	/	7.86	2.31	30%
C0	200	300	/	/	5.99	1.68	40%
BV1	150	350	/	2.5	7.86	2.31	30%
BV2	150	350	/	5.0	7.86	2.31	
BV3	150	350	/	10.0	7.86	2.31	
BM1	150	350	5	/	7.86	2.31	
BM2	150	350	10	/	7.86	2.31	
BM3	150	350	15	/	7.86	2.31	

### Testing methods

The roundness and sphericity of the as-prepared samples were performed by a stereomicroscope (Shenzhen Aosvi Microoptical Instrument Co., Ltd). The phase analysis of proppants products was investigated by XRD. The microstructure of the proppant specimens before and after the acid treatment was observed by scanning electron microscopy (SEM, Gemini 300, Zeiss-Jena Model). The acid treatment was implemented according to the Chinese standard “SY/T 5108-2014”. The acid solution was first pre-fabricated as HCl:HF = 12:3 (mass 12% HCl and mass 3% HF). Afterward, the ceramic proppant was totally immersed in the acid solution which was maintained at a constant temperature of 66 °C for 3 h to remove the soluble substances from the ceramic proppant. The mass changes and thermodynamic behavior of samples were performed using a thermogravimetric analyzer and differential scanning calorimetry (TG-DSC, NETZSCH, STA449F3) at a heating rate of 10 °C/min and an operating temperature range of room temperature to 1400 °C. Furthermore, the bulk density, apparent density, roundness, sphericity, acid solubility and breakage ratio (52 MPa), and acid solubility of the proppant were all tested following the oil and gas industry standard SY/T 5108-2014, respectively. The properties of sintered proppants should meet the requirements of SY/T 5108-2014 standard “Proppant performance test method for hydraulic fracturing operations”. This standard indicated a comprehensive assessment of the proppant performance, including the safety and environmental control requirements in hydraulic fracturing operations. It stipulated that the crushing rate of the 7.5 K ceramic proppant should not exceed 9% under a closing pressure of 52 MPa, and the acid solubility should be limited to no more than 7%.

## Results and discussions

### The influence of sintering temperature and ODCPRs contents on the performance of the proppants

#### Performance test

To determine the effect of sintering temperature and ODCPRs contents on the physical–mechanical performance of proppants and optimize the sintering conditions, proppants were prepared with various ratios of ODCPRs and sintered at different temperatures. The physical and mechanical performances of the proppants, including the bulk density, apparent density, roundness, sphericity, acid solubility and breakage ratio, and acid solubility were analyzed.

**Figure 1:**
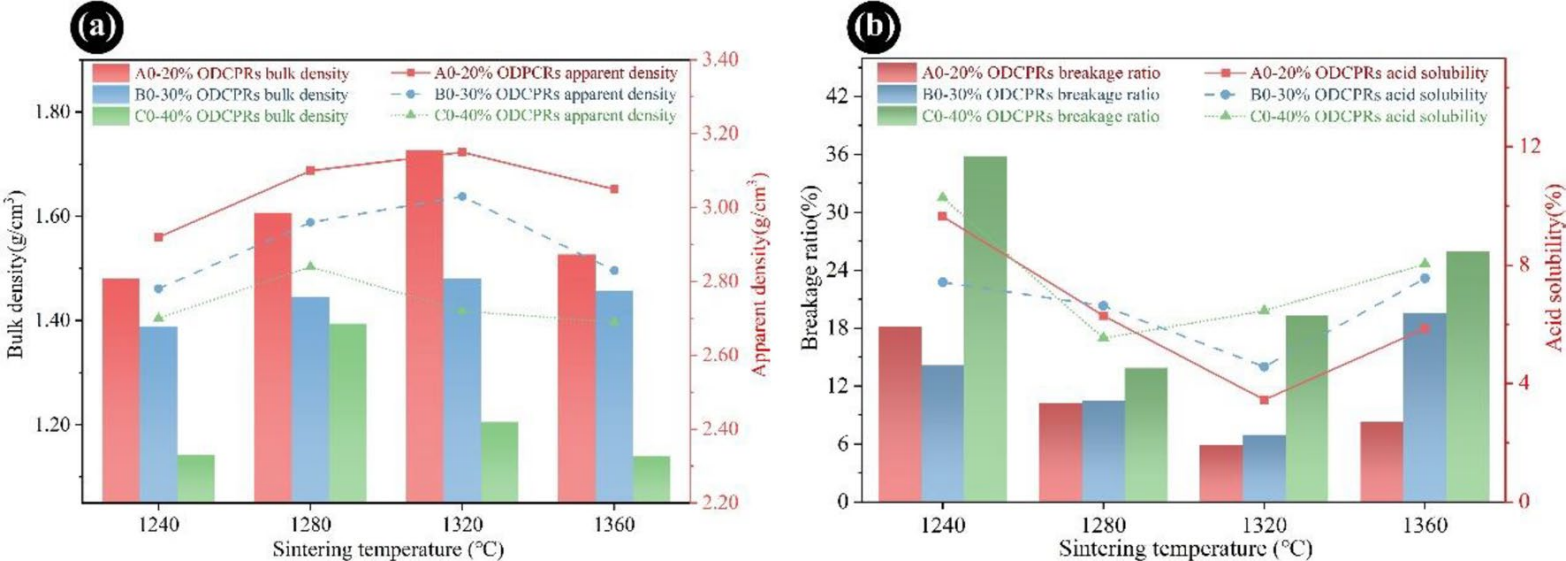
The properties of samples with different contents of ODCPRs sintered at various temperatures: (**a**) bulk density and apparent density; (**b**) breakage ratio and acid solubility.

As displayed in Fig. 1a,b, when the ODCPRs contents were improved from 20 to 40%, the bulk density and apparent density of proppants decreased in all sintering temperature points, and the breakage ratio showed the opposite tendency. Whereas the acid solubility of the proppants has fluctuated, which was not only impacted by the ODCPRs contents but also the sintering temperature. The acid solubility was closely correlated to the soluble phases in the proppant, and a large amount of alkaline earth metal oxides was significantly improved with the increment of ODCPRs (such as potassium, sodium, and calcium oxide), which would facilitate the formation of liquid phases under a higher sintering temperature. Thus, under the condition of a relatively high temperature or high ODCPRs dosage, more glass phases would be generated, attributing to a higher acid solubility.

Furthermore, with the increment of sintering temperatures, the optimal sintering temperatures of the A0, B0, and C0 formulas with their lowest breakage ratio and acid solubility all first declined and raised subsequently. The best sintering temperatures of the three formulas could be confirmed as 1320 °C, 1320 °C, and 1280 °C, respectively. According to Table 1, with the increase of the ODCPRs content, both the K value and the molar ratio (Al_2_O_3_/SiO_2_) in initial mixtures were significantly reduced, indicating that the content of flux oxides and silica in the formula was also gradually improved, thus the sintering temperature declined at the higher ODCPRs content. Therefore, formulas of A0 and B0 at 1320 °C had exhibited better performance. Noteworthy, the ODCPRs consumption and the cost of raw materials should be comprehensively considered as well, hence formula B0 sintering at 1320 °C was identified as the optimal choice with the lowest breakage ratio (6.97%) and acid solubility (4.56%).

**Figure 2:**
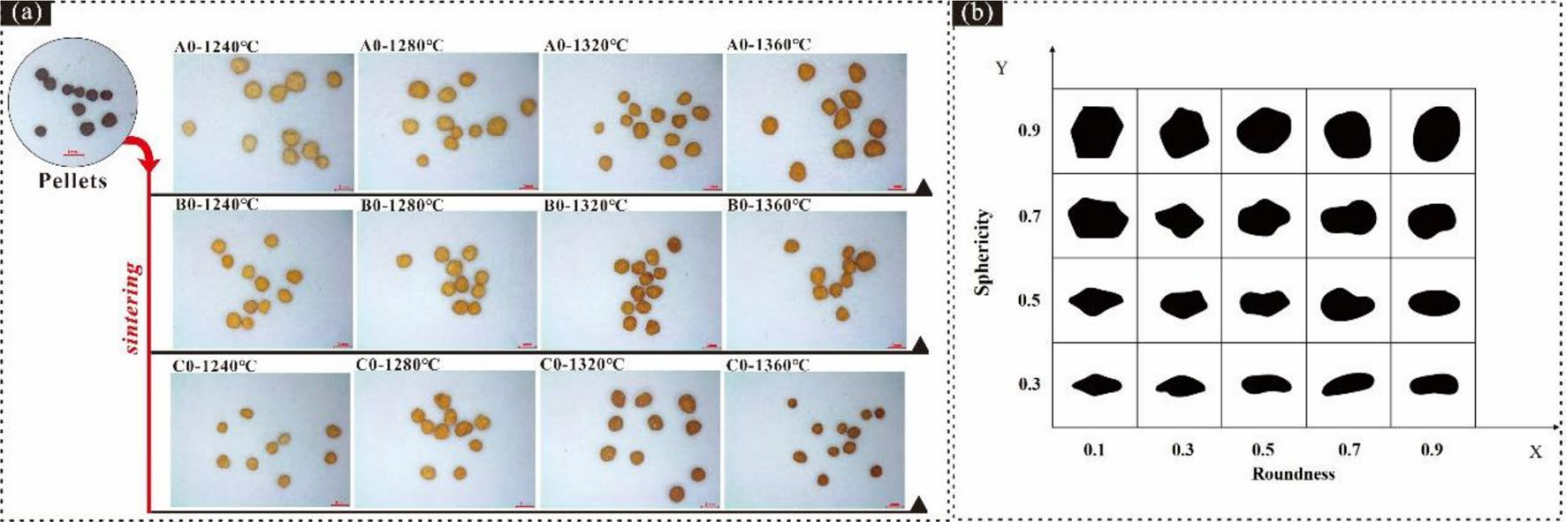
(**a**) Photograph of proppants under different sintering temperatures; (**b**) the Krumbern/Sloss template.

Additionally, the photographs of proppants produced in the experiment were displayed in Fig. 2a, it could be observed that the color was darkened as the ODCPRs content or sintering temperature increased. Besides, the Krumbein/Sloss template was used to evaluate the roundness and sphericity of the proppants as shown in Fig. 2b. The average roundness and sphericity degree of all proppants (A0–C0) was above 0.8, which could well meet the requirements of the oil and gas industry standard SY/T 5108-2014.

#### Phase analysis

**Figure 3:**
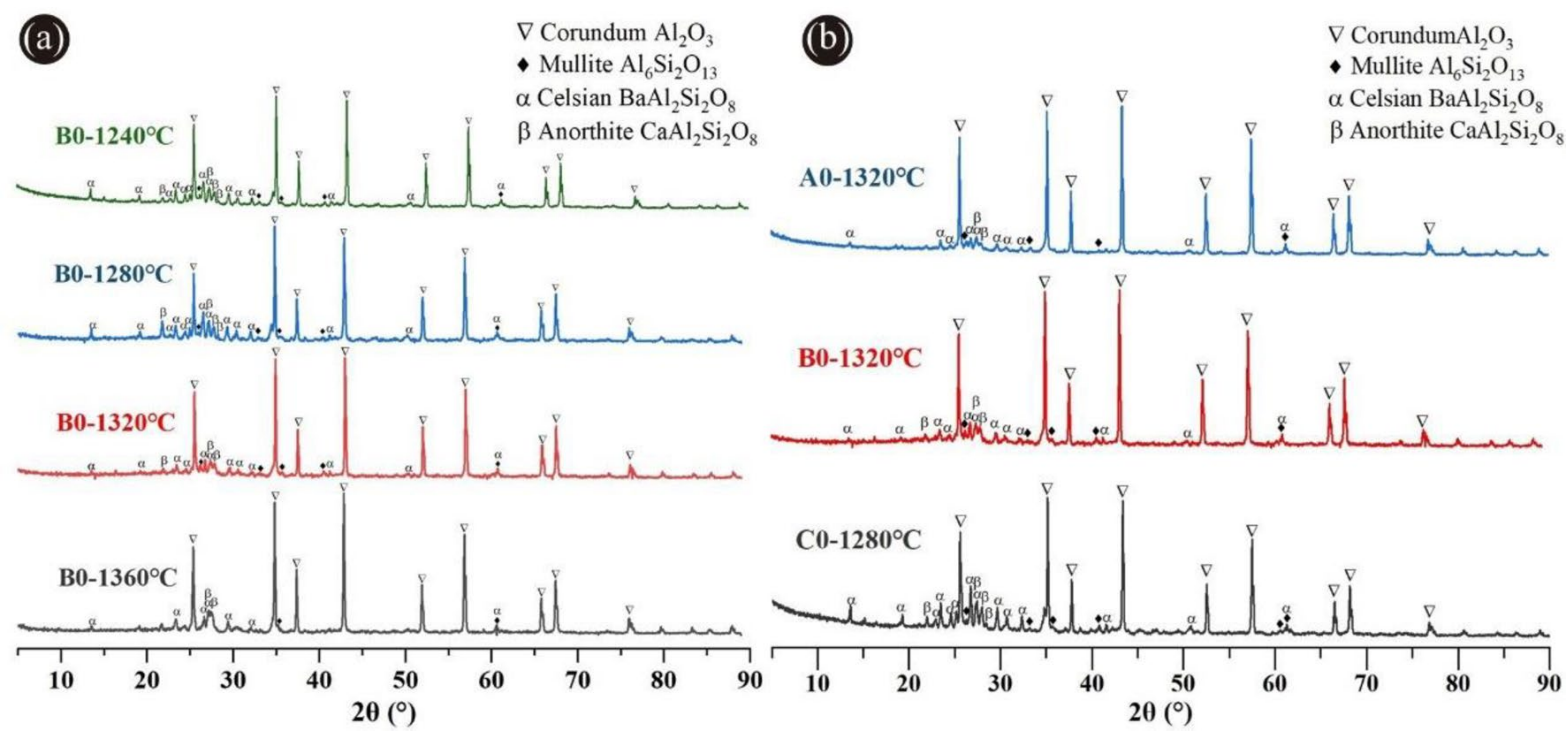
XRD patterns of ceramic proppants: (**a**) formula B0 sintered at different temperatures, (**b**) formulas with different ODCPRs contents at their optimum sintering temperatures.

Figure 3a showed the XRD patterns of ceramic proppants of the B0 formula in different sintering temperatures. It can be observed that the crystalline phases of these proppants mainly consisted of corundum and a small quantity of mullite, celsian, and anorthite. Corundum was the predominant crystalline phase that was ascribed to the initial mixtures with a high molar ratio (Al_2_O_3_/SiO_2_), resulting in a highly conducive to the generation of the corundum phase. The diffraction peak of the mullite crystal was weak following that mullite has a relatively low content in this sintering temperature range. Corundum with high crystallinity and granulate mullites can improve the toughness of the ceramic proppants attributing to the reduction of breakage ratio^28,32^. Thus, as the sintering temperature increased from 1240 to 1360 °C, the diffraction peak intensities of corundum and mullite crystals both enhanced, which would facilitate the development of high-performance ceramic proppants. Nonetheless, owing to masses of metallic oxides in the ODCPRs, some celsian and anorthite were also generated by the reaction between the BaO, CaO, SiO_2_ in the ODCPRs, and Al_2_O_3_ in bauxite. After the sintering temperature raised over 1320 °C, the intensity of the celsian and anorthite phases' diffraction peaks was weakened, which could be because the celsian and anorthite phases gradually changed from the solid to the glass phase with the temperature ascending. But too many liquid phases would eventually exist in the amorphousness of the proppants, contributing to a negative influence on the performance of proppants^23^.

Additionally, as shown in Fig. 3b, when the ODCPRs content was improved, both the intensities of corundum and mullite diffraction peaks were reduced but the celsian and anorthite phases` diffraction peaks were enhanced at their respective optimal sintering temperature. It was implied that the crystallinity of the corundum and mullite phases decreased with the reduction of the molar ratio (Al_2_O_3_/SiO_2_), while the excessive Ba^2+^ and Ca^2+^ in the matrix reacted with Al and Si that formed the celsian and anorthite phases. Conversely, the formation of excessive celsian and anorthite phases might inhibit the development of corundum and mullite phases, and remarkably impair the performance of proppants^33^. Thereby, the liquid phase was also increased owing to the augment of ODCPRs content, causing the descent of the sintering temperature, such as the formula C0.

#### Morphology analysis

Figure S4 displayed the cross-sectional microscopic morphology of the proppants before the acid treatment of formula B0 under different sintering temperatures. With the increase of the sintering temperatures, the prevalence of the inner pores initially decreased and then turned to increase. A loose internal structure with less molten phase and high porosity was observed in the proppant when the optimal sintering conditions were not satisfied, as shown in Fig. S4a. When the sintering temperature gradually improved, a denser structure was achieved, on account of more liquid phases filling in the pores, as exhibited in Fig. S4b,c, and the molten phases also formed a cohesion bonding between the crystal particles and strengthened physical and mechanical properties of ceramic matrix^34^. Howeveduer, as demonstrated in Fig. S4d, when the sintering temperature was increased over 1360 °C, more elongated and connected pores appeared in the interior, which was attributed to generating the excessive liquid phase and promoting the expansion of the ceramic body, resulting in the decrease in density and strength of proppants^35,36^.

**Figure 4:**
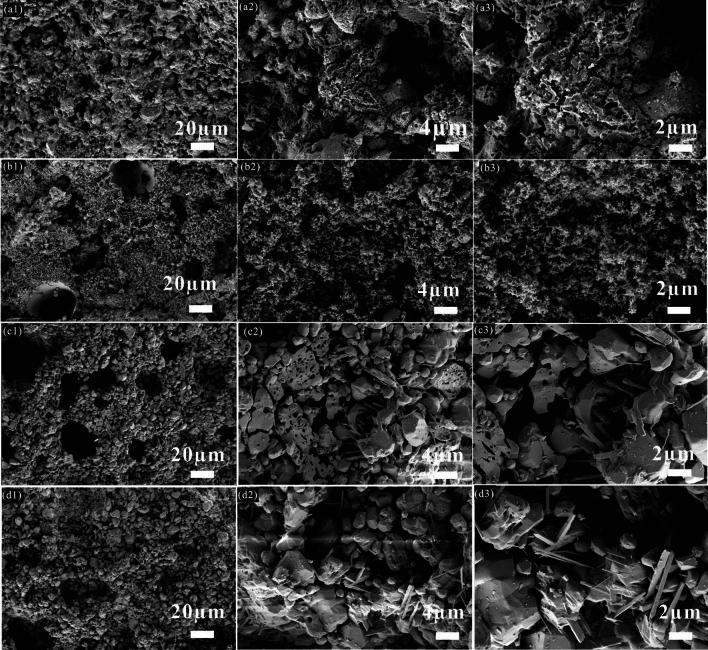
Microscopic cross-sections of the ceramic proppant after the acid treatment of the formula B0 at different sintering temperature: (**a**) 1240 °C; (**b**) 1280 °C; (**c**) 1320 °C; (**d**) 1360 °C.

As shown in Fig. 4, the inner appearances of the proppants of formula B0 at different sintering temperatures were also investigated after the acid treatment. Compared to the results from Fig. S4, it can be indicated that the whole equilibrium network structure of the proppant matrix was comprised of the residue glass phase and crystal phases. As seen from Fig. 4a, when the proppant was calcined at 1240 °C, the after-eroded net-like phases scattered around the incomplete sintered granular crystal particles. With increasing the sintering temperature to 1280 °C, the sample was mainly formed by abundant dispersive small corundum grains with loose internal structures, as shown in Fig. 4b. Subsequently, when the sintering temperature reached 1320 °C, as presented in Fig. 4c,d, interlocking columnar and granular shapes of crystals occurred, which were corresponding to the growth of the mullite and corundum crystals. Furthermore, the crystals became bigger under a relatively high temperature (1360 °C), which illustrated that a proper sintering temperature would enhance the development of corundum and mullite phases. Good growth of corundum has the advantages of high hardness, strength, and resistance to chemical erosion^37^, thus the proppants of formula B0 at a relatively higher sintering temperature showed better performance.

**Figure 5:**
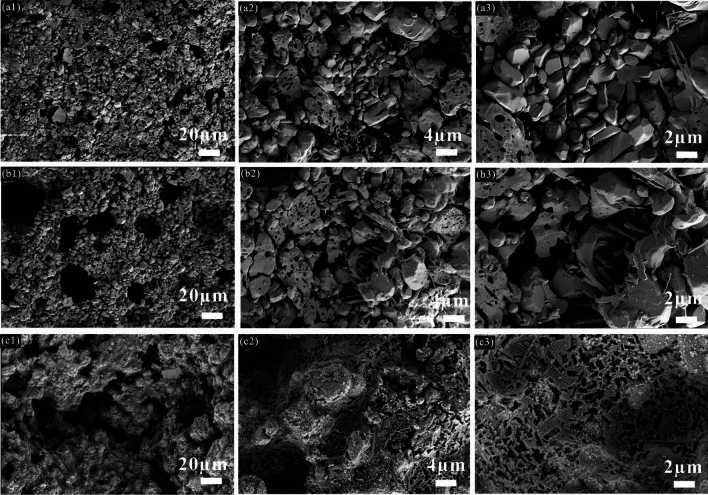
Microscopic cross-sections of the ceramic proppants after the acid treatment with different ODCPRs contents at their optimum sintering temperature: (**a**) A0 at 1320 °C; (**b**) B0 at 1320 °C, (**c**) C0 at 1280 °C.

Figure S5 displayed the microscopic morphology of the proppants with different ODCPRs contents at their optimum sintering temperature, respectively. Three formulas of the proppants were all well-sintered, meanwhile obvious porous inner structures occurred in all samples as well. Afterward, with the increase of ODCPRs content, more liquid and gas phases were generated during the sintering process. These gas phases were produced from mineral decompositions, leading to an increase in both the number and size of the inner pores of the samples, such as CaCO_3_ and BaSO_4_. In parallel with this, the inner crystal appearance of samples with different formulas was also studied after the acid treatment, as displayed in Fig. 5. The results further elaborated that a denser structure composed of granular corundum and rod-like mullite phases was achieved when enough content of Al_2_O_3_ and molar ratio (Al_2_O_3_/SiO_2_) was provided, as shown in Fig. 5a,b. However, when ODCPRs contents of the proppant improved beyond 40%, too many ODCPRs in formula C0 formed too much glass phase, as shown in Fig. 5c, causing a relatively higher breakage ratio and acid solubility.

### The influence of holding time on the performance of the proppants

#### Performance test

**Figure 6:**
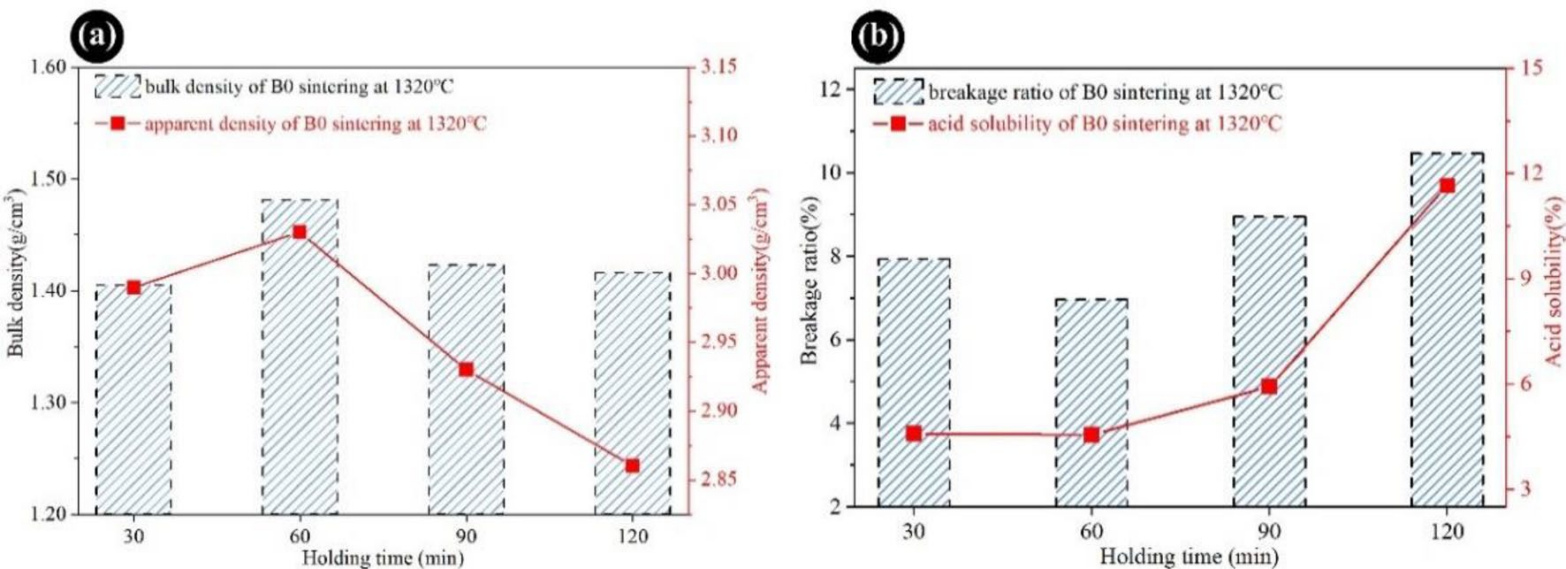
The properties of B0 samples sintered at different holding times: (**a**) bulk density and apparent density; (**b**) breakage ratio and acid solubility.

To guarantee the transition of ceramic proppants into a compacter structure through the sintering reaction, it is necessary to explore the effect of holding time on the performance of proppants. As presented in Fig. 6a, the bulk density and apparent density of proppants under different holding times at 1320 °C were carried out. The bulk density and apparent density of proppants exhibited the same tendency which was first increased and then decreased observably with the increase of the holding time, the biggest bulk density and apparent density were obtained at 1320 °C and heat preservation for 60 min, as 1.48 g/cm^3^ and 3.03 g/cm^3^, respectively. Furthermore, the breakage ratio and acid solubility of proppants initially decreased and gradually raised with the extension of the holding time, as shown in Fig. 6b. The lowest breakage ratio (6.97%) and acid solubility (4.56%) of the proppants were acquired by heating preservation for 60 min, which could well meet the standard of SY/T 5108-2014. Thus, guaranteeing a proper holding time during the calcination process for preparing ODCPRs-based ceramic proppants at the best sintering temperature was beneficial.

#### Phase analysis

**Figure 7:**
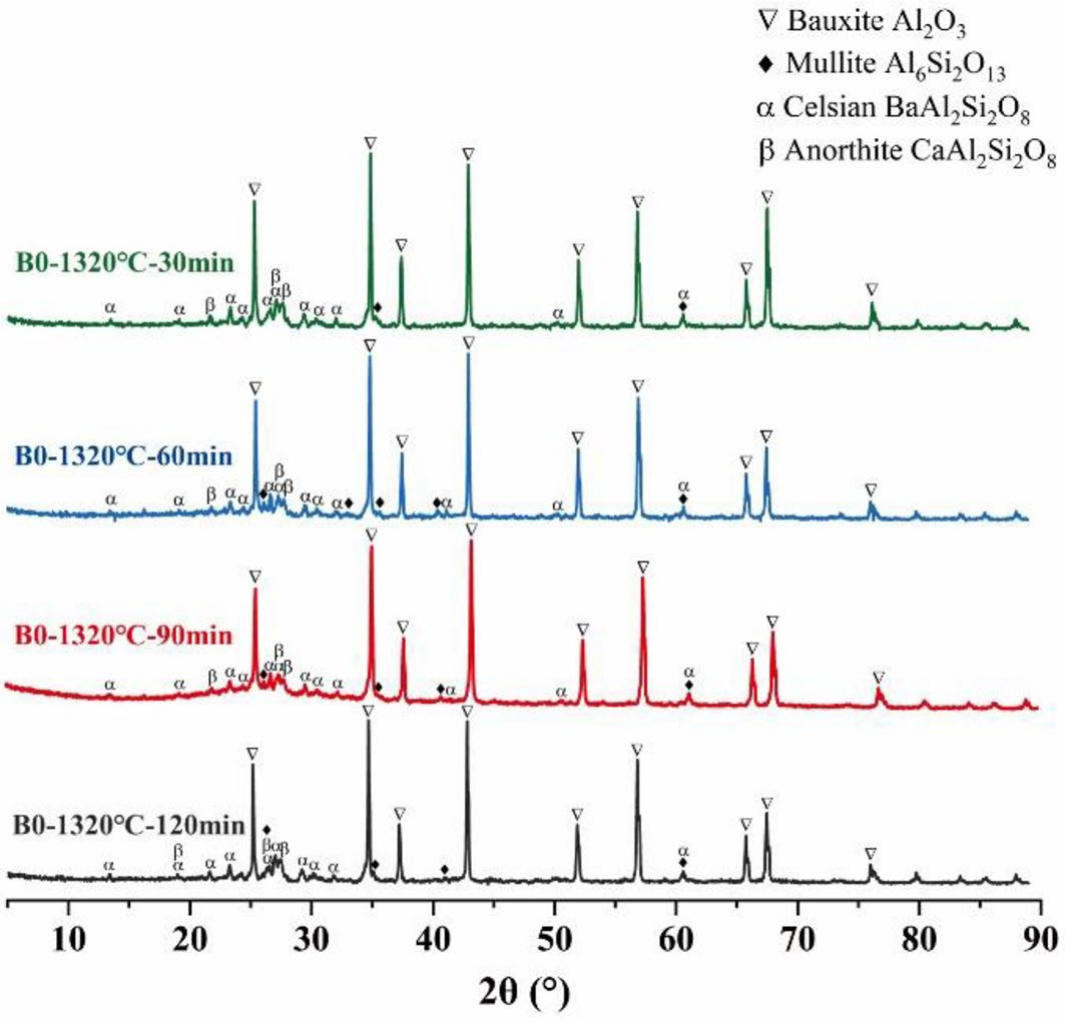
The XRD patterns of B0 samples sintered at different holding times.

The XRD patterns of formula B0 under different holding times were also shown in Fig. 7, and the modest heat preservation (holding for 60 min) was advantageous to the formation and growth of corundum and mullite crystals. Meantime, it also resulted in the reduction of bauxite and celsian phases. However, the increase in holding time had no obvious influence on the final phase category of the proppant, which deduced that the variation in the performances of the proppant was mainly caused by the microstructure and glass phase content changes of the ceramic proppant.

#### Morphology analysis

**Figure 8:**
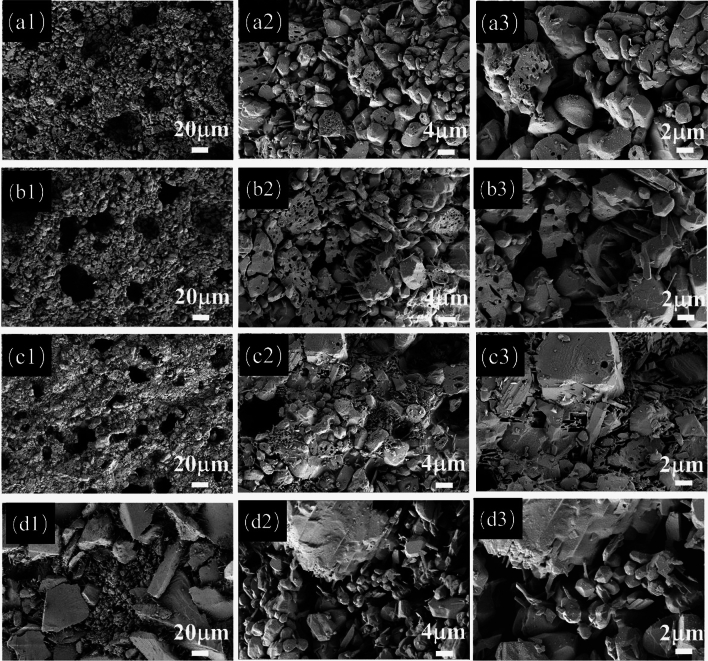
Microscopic diagram of the ceramic proppant after the acid treatment of the formula B0 sintered at different holding times: (**a**) 30 min; (**b**) 60 min, (**c**) 90 min; (**d**) 120 min.

Figures S6 and 8 displayed the microscopic diagrams of the ceramic proppant before and after the acid treatment of the formula B0, which were sintered with different holding times at 1320 °C, respectively. As observed in Fig. S6, with the extension of holding time, the pore characteristics of the proppants before acid treatment were similar. Nevertheless, after the elimination of the glass phase, the microstructure of the crystals resulted in obvious differences. When the holding time was only 30 min, plentiful coarse granular corundum grains and a small amount of needle-shaped mullite were observed. After the holding time was improved to 60–90 min, both the corundum and mullite crystals grew bigger (Fig. 8a,b). When the holding time reached 120 min, some coarse granular corundum crystals grow up into big particles, which were with excessive growth and an average grain size of about 20 μm. Over the holding time, the abnormal excessive growth of grains would be adverse to gas exhaustion and easily form internal stress failure, which manifested as a change in density and decrease in porosity and strength^38,39^. Thus, uniformly distributed and smaller crystals improved the mechanical performance of ceramic proppants better.

### The influence of additives on the performance of the proppants

#### Performance test

Metallic oxide sintering additives were used to promote the liquid phase formation and the liquid viscosity alteration of the ceramic system, which can significantly improve the performance of proppants^40,41^. In this section, the performances of ODCPRs ceramic proppants with different mass fractions of V_2_O_5_ and MnO_2_ additives at various sintering temperatures were studied.

**Figure 9:**
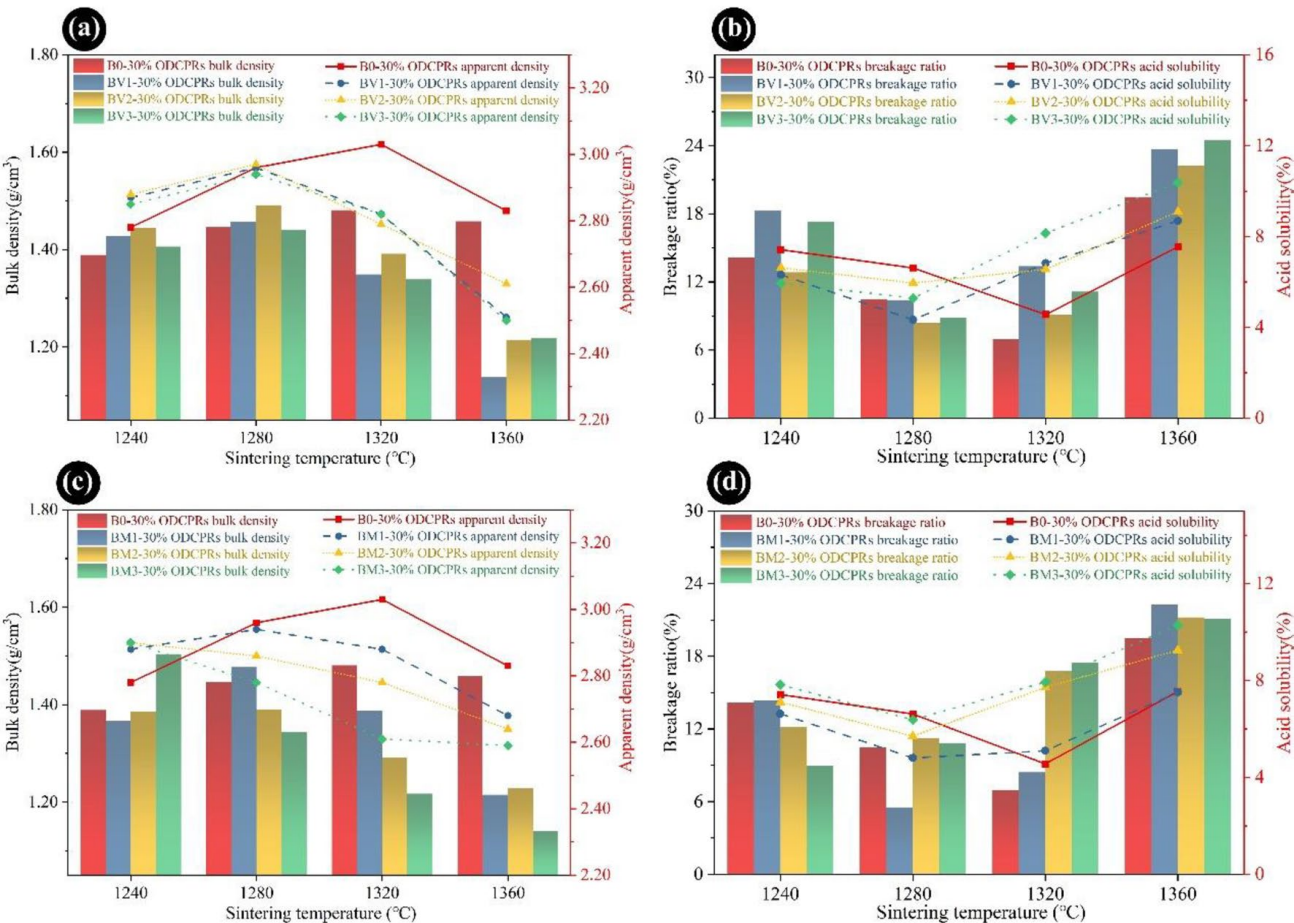
The properties of proppant samples with different dosages of V_2_O_5_ and MnO_2_ additives sintered at various temperatures: (**a**,**c**) bulk density, and (**b**,**d**) breakage ratio.

As plotted in Fig. 9a, when the sintering temperature was below 1280 °C, with an increase in V_2_O_5_ dosage, the bulk density and apparent density of formula B0 were first increased slightly and then decreased significantly. Moreover, the density of proppants turned out to be much lower when the sintering temperature rose beyond 1320 °C. Furthermore, the whole series of BV samples showed a distinct decrease in breakage ratio and acid solubility when the sintering temperature reached 1280 °C, as displayed in Fig. 9b. Thereinto, sample BV2 presented the minimum value of breakage ratio (8.40%), and sample BV1 presented the minimum value of acid solubility (4.33%). Although the sintering temperature of the proppant was reduced after the incorporation of V_2_O_5_ additives that could reduce the energy consumption, the results also elucidated that the mechanical and acid resistance performance was still undesirable in comparison with sample B0 under its optimum sintering condition. This was ascribed to the promotion effect of the sintering reaction, because of the addition of V_2_O_5_, which led to more densification of the proppants with an appropriate amount of liquid phase. However, when under high-temperature conditions, too many liquid phases formed^42^, while O_2_ from the decomposition of V_2_O_5_ would also happen at a high temperature, finally resulting in the decline of the mechanical performance of the proppant^42^.

Figure 9c showed the bulk density and apparent density of formula B0 with different dosages of MnO_2_. With the increase in the MnO_2_ dosage, the sintering temperature and the density showed a descending tendency, indicating that a low-temperature liquid phase was formed during the sintering process, on account of the excellent sintering ability of MnO_2_^27,41^. Simultaneously, variations of breakage ratio and acid solubility with the sintering temperature of proppants were shown in Fig. 9d. It can be found that the breakage ratio and the acid solubility of the proppants decreased with a sintering temperature up to 1280 °C and followed by an increase with a sintering temperature up to 1320 °C. The minimum breakage ratio and acid solubility was achieved by formula BM1 as 5.25% and 4.80% at 1280 °C, respectively, which was much better than formula B0.

#### Phase analysis

**Figure 10:**
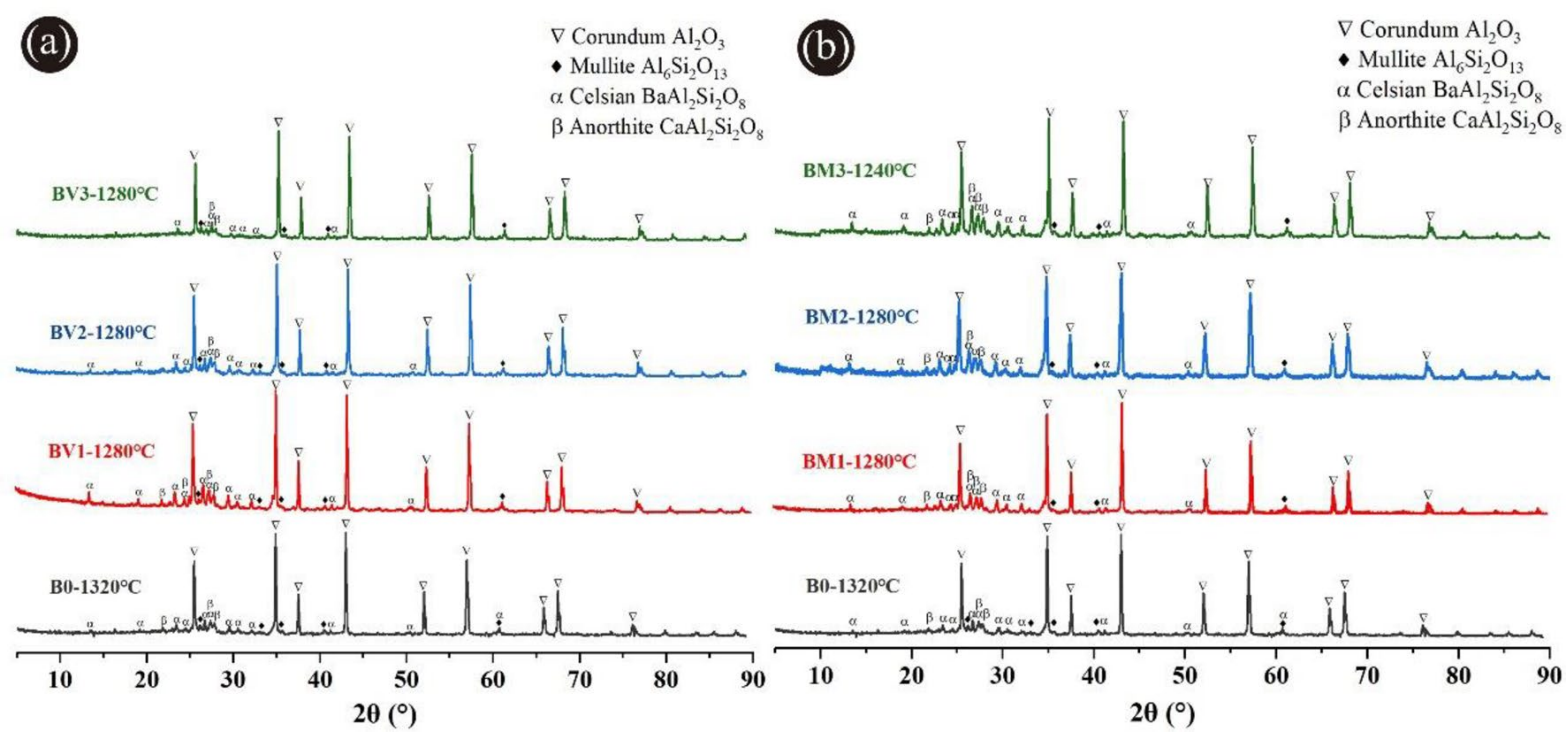
XRD patterns of ceramic proppants under different sintering temperatures added with (**a**) V_2_O_5_ additives and (**b**) MnO_2_ additives.

Figure 10 gave the XRD patterns of proppant samples added with different contents of V_2_O_5_ and MnO_2_ at their optimum temperatures, indicating there were no new diffraction peaks detected in the crystalline phases in the product after the addition of V_2_O_5_ or MnO_2_. It also can be observed from Fig. 10a, the diffraction peak intensity of crystal phases has no obvious change with the augment of the V_2_O_5_ additive dosage. This implied that the addition of V_2_O_5_ additives has no observable influence on the phase composition of the proppant, which would be attributed to V_2_O_5_ easily entering the lattice of mullite crystals as a solid solution and promoting the formation of the liquid phase during the high-temperature sintering process^43–46^.

According to Fig. 10b, the peak intensity of corundum and mullite phases of samples containing MnO_2_ additives were very similar to formula B0. The reason was Mn^4+^ was able to dissolve into the corundum phase and replace Al^3+^ to form a finite solid solution during the sintering process, which caused the lattice distortion and promotion of grain growth, reduction of sintering temperature, and the densification of structure^27,36,41,47^. Whereas, the peak intensity of anorthite and celsian was enhanced with the increase of the MnO_2_ additive, indicating the addition of MnO_2_ could facilitate the generation of the anorthite and celsian. The formation of celsian and anorthite was related to the abundant BaO and CaO in the ODCPRs, and the excessive glassy phase was generated owing to the fluxing effect of the MnO_2_ additive could further stimulate the growth of celsian and anorthite^48,49^.

#### Morphology analysis

Figures S7 and S8 showed the cross-sectional microscopic images of the proppants with different dosages of V_2_O_5_ and MnO_2_ after the acid treatment, severally. The results from Fig. S7 revealed that the addition of V_2_O_5_ has a remarkable influence on the formation of mullite. The sample without V_2_O_5_ only contained a few needle-like mullite crystals (Fig. S7a). As the dosage of V_2_O_5_ increased within the range of 0.5–1 wt%, anisotropic mullite crystals in significant amounts appeared among the granular corundum particles (refer to Fig. S7b,c) which contributed to enhancing the mechanical strength and acid resistance of the ceramic proppant. With further improvement of the V_2_O_5_ dosage, the needle-like mullite crystals changed into bigger rod-like mullite (Fig. S7d). This denoted that V_2_O_5_ additives can dramatically drive the in-situ development of spearhead columnar mullite, on account of the increase of liquid phase in the reaction system, which was corresponding to the analysis of XRD results.^42^, which was corresponding to the analysis of XRD results.

The microscopic diagrams of the ODCPRs proppants with various MnO_2_ dosages were exhibited in Fig. S8. The observation of corundum phases in samples containing MnO_2_ was consistent with the B0 sample. Multiple corundum grains were gathered together to form a granular shape, while only a small number of acicular mullite was distributed among the corundum grains, indicating that the addition of MnO_2_ does not influence the growth of mullite crystals but promoted the development of corundum. Noteworthy, as shown in Fig. S8b, at the MnO_2_ dosage of 2 wt%, the cluster shape of celsian was observed, which was in accordance with the results of XRD. With further increase in the MnO_2_ dosage at 3 wt%, the liquid phase dramatically increased and gradually englued the crystal grains, and some incompleted porous glassy phase dispersed in the matrix after the acid treatment, as the remnants left after being corroded that would seriously affect the mechanical strength and density of the ceramic proppants.

### Thermal behaviors analysis

**Figure 11:**
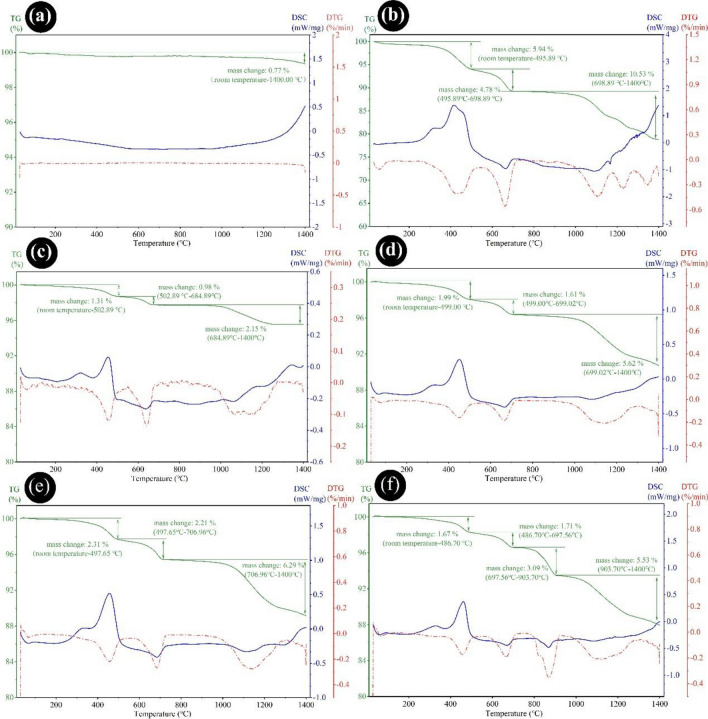
TG-DSC curves of samples (**a**) bauxite; (**b**) ODCPRs; (**c**) A0; (**d**) B0; (**e**) C0; and (**f**) BM1.

The mass change and sintering behaviors of the raw materials were comprehensively evaluated through TG-DSC experiments. As depicted in Fig. 11a,b, the pure bauxite has presented negligible weight variation during the calcination process, indicating a relatively stable chemical structure. However, an exothermic phenomenon occurred above 1200 °C, leading to the recrystallization of Al_2_O_3_ at high temperatures. Meanwhile, as shown in Fig. 11b, distinct weight reduction stages were observed at 25–495 °C, 495–698 °C, and 698–1400 °C for the ODCPRs, which corresponded to three different sintering processes: firstly, when the heating temperature was raised from room temperature to 495 °C, a primary weight loss of 5.94% occurred according to the evaporation of free, absorbing and crystal water as well as combustion of residue organic constituents (such as oil)^50^. Subsequently, a steep weight loss of 4.78% occurred when the temperature was above 698 °C that corresponded to an obvious endothermic peak (664 °C), which would be attributed to the violent decomposition of carbonates^51,52^. Thirdly, an dramatic weight quality loss of 10.53% occurred from 698 °C to 1400 °C, and an endothermic peak (1167 °C) was observed, which might be related to the solid-phase reaction, on account of the generation of a liquid phase or the transformation between crystal forms of salt substances at high temperatures (such as the decomposition of BaSO_4_)^53,54^.

The thermal behavior of the mixtures with 20 wt%, 30 wt%, and 40 wt% ODCPRs were displayed in Fig. 11c–e respectively, which have shown a similar weight descending trend with the pure ODCPRs sample, and all samples included three different weight loss stages. The results suggested that with the increase of the ODCPRs content, greater weight loss of the samples can be achieved. Meantime, the appearance of endothermic peaks was also delayed because more energy was consumed for thermal decomposition reactions. Specifically, the most significant increase in weight loss occurred during the solid-phase reaction process, and the greatest mass loss (6.32%) was generated by C0, which may be attributed to the formation of more molten phases that accelerated the growth of corundum.

Moreover, the thermal behavior of BM1 was demonstrated in Fig. 11f, with the addition of MnO_2_ powders, a new weight loss stage emerged between 697 and 903 °C. This could be attributed to the decomposition of MnO_2_ powders, which act as a sintering aid in the raw material and induce molten action between the solid phase within the high-temperature range^40^. Combined with Fig. 11b, no new phase formation has been observed following the incorporation of MnO_2_. This phenomenon can be ascribed to the increased formation of the liquid phase at high temperatures, which was caused by the melting of aluminosilicate, on account of the distortion of Mn^2+^ into the corundum lattice and solid solution formation^41^. This further promoted the liquid–solid reactions, resulting in a greater mass change within the 903–1400 °C stage. Consequently, the proppant density and strength were enhanced by optimizing and shrinking the interface among solid phases through the addition of MnO_2_. Based on the preceding discussions, the proppant underwent various reactions during sintering, including water evaporation, hydrocarbon combustion, carbonate and sulfate decomposition, glass phase melting, and corundum recrystallization, as follows^41,52–56^:

1 $${\text{H}}_{{2}} O(l)\mathop{\longrightarrow}\limits^{{100 - 200\;^\circ {\text{C}}}}H_{2} O \uparrow $$

2 $$CxHy({\text{hydrocarbon}}) + O_{2} \mathop{\longrightarrow}\limits^{200 - 600^\circ C}CO_{2} \uparrow + H_{2} O \uparrow$$

3 $$CaCO_{3} \mathop{\longrightarrow}\limits^{{650 - 750\;^\circ {\text{C}}}}CaO + CO_{2} \uparrow$$

4 $$Al_{2} O_{3} ({\text{pseudocorundum}})\mathop{\longrightarrow}\limits^{{900 - 1000\;^\circ {\text{C}}}}Al_{2} O_{3} ({\text{corundum}})$$

5 $$ BaSO_{3} + RO_{x} ({\text{metallic oxide}})\mathop{\longrightarrow}\limits^{{1000 - 1350\;^\circ {\text{C}}}}BaRO_{y} + SO_{2} \uparrow $$

6 $$ MnO_{2} \mathop{\longrightarrow}\limits^{{460 - 570\;^\circ {\text{C}}}}Mn_{2} O_{3} \mathop{\longrightarrow}\limits^{{926\;^\circ {\text{C}}}}Mn_{3} O_{4} \mathop{\longrightarrow}\limits^{{1158\;^\circ {\text{C}}}}MnO$$

7 $$2MnO\mathop{\longrightarrow}\limits^{{Al_{2} O_{3} }}2Mn_{{_{Al} }}^{`} + V_{O}^{``} + 2O_{O}^{X}$$

## Conclusion

In summary, this work explored a new research method for preparing high-performance proppants from ODCPRs, bauxite, and sintering additives. To optimize the performance of proppants, variations in the mass ratio of ODCPRs, sintering temperature, holding time, and additives were studied in detail. Finally, the thermal behavior and the sintering reactions of the proppant were stated.

The results illustrated that the proppant with ODCPRs underwent various reactions during sintering, including water evaporation, hydrocarbon combustion, carbonate and sulfate decomposition, glass phase melting, and corundum recrystallization. Thereinto, a higher content of liquid phases in proppants would be generated with the increase of sintering temperature and the ODCPRs content, and abnormal excessive growth of crystal grains would also happen over the holding time, attributing to the decrease in strength and acid solubility. Meanwhile, the main phases in all formulas were corundum and a small quantity of mullite, celsian, and anorthite, caused by the variation of the sintering temperature or holding time just led to the change in phase morphology and phase content, which still seriously impacted the property of proppants. Furthermore, the addition of sintering additives would promote the formation of corundum or mullite crystal phases and molten phases at the same time, which was the primary source of high strength and densification of proppants. But excessive liquid phases and growth of the crystal in proppant would form when the additives increase at a higher level, contributing to a negative influence on the performance of proppants. Therefore, at the best sintering condition (1280 °C, holding for 60 min) and a mass ratio (ODCPRs:bauxite:MnO_2_ at 3:7:0.1), the well-developed granular corundum and acicular mullite formed inside the proppants and interspersed with each other to form a dense structure. The proppants presented low density and high strength as the bulk density of 1.48 g/cm^3^, the apparent density of 2.94 g/cm^3^, a breakage ratio of 5.25% under 52 MPa closed pressure, and acid solubility of 4.80%, which could well meet the requirement of the standards of SY/T 5108-2014.

### Supplementary Information

Supplementary Information.

## Data Availability

The data are available from the corresponding author on reasonable request.
