# Supplementary material for: Preparation and characterization of high-performance ceramic proppant from recycling utilization of oil-based drilling cuttings pyrolysis residues
Source: Sci Rep. 2024 Jan 29;14:2345. doi: 10.1038/s41598-024-52334-7 (PMC11222615; doi:10.1038/s41598-024-52334-7)
Supplement: Supplementary file 1 — Supplementary Information. [file 41598_2024_52334_MOESM1_ESM.docx]

**Supporting information for**

**Preparation and characterization of high-performance ceramic proppant from recycling utilization of oil-based drilling cuttings pyrolysis residues**

Yuanyi Yang^a, d, 1*^, Hui Li^b, c, 1^, Zhenghuan Lei^a^, Hongwu Liu^a^, Mingyou Zeng^a^, Tingting Yang^a^, Keming Chen^b^, Yi Duan^b^

^a^ School of Civil Engineering and Geometrics, Southwest Petroleum University, Chengdu, 610500, China

^b^ CNPCCCDE, Safety Environment Quality Surveillance & Inspection Research Institute, Guanghan, 618000, China

^c^ College of Environmental Science and Engineering, China West Normal University, Nanchong, 637009, China

^d^ Research Institute of Engineering Safety Assessment and Protection of Southwest Petroleum University, Chengdu, 610500, China

*Corresponding author:

A/Prof. Yuanyi Yang,

School of Civil Engineering and Geometrics, Southwest Petroleum University;

E-mail: [yangyuanyi@swpu.edu.cn](mailto:yangyuanyi@swpu.edu.cn)

^1^These authors contributed equally to this work.

**Captions of figures and tables**

**1. Fig. S1.** The XRD patterns of raw materials: (a) ODCPRs, (b) bauxite.

**2.** **Fig. S2.** Preparation procedure of ceramic proppants.

**3. Fig. S3.** (a) The particle size distribution of the grounded raw material, (b) the sintering systems of ceramic proppants with ODCPRs.

**4. Fig. S4.** Microscopic diagrams of the ceramic proppant before the acid treatment of the formula B0 at different sintering temperatures: (a) 1240℃; (b) 1280℃; (c) 1320℃; (d) 1360℃.

**5. Fig. S5.** Microscopic diagram of the ceramic proppants before the acid treatment with different ODCPRs contents at their optimum sintering temperature: (a) A0 at 1320℃; (b) B0 at 1320℃, (c) C0 at 1280℃.

**6. Fig. S6.** Microscopic diagram of the ceramic proppant before the acid treatment of the formula B0 sintered at 1320℃ with different holding times: (a) 30min; (b) 60min, (c) 90min; (d) 120min.

**7. Fig S7.** Microscopic diagram of the ceramic proppant with different V_2_O_5_ additives after the acid treatment sintered at 1280℃: (a) B0 (b)BV1; (c) BV2; (c) BV3.

**8. Fig S8.** Microscopic diagram of the ceramic proppant with different MnO_2_ additives after the acid treatment sintered at 1280℃: (a) BM1; (b) BM2; (c) BM3.

**9.** **Table S1.** The chemical composition of the raw materials.

**
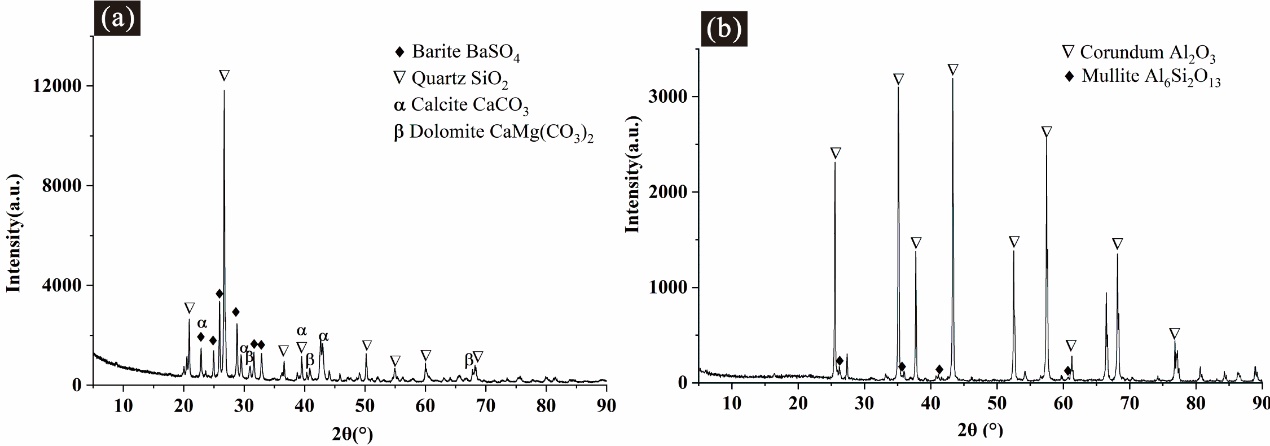
**

**Fig. S1.** The XRD patterns of raw materials: (a) ODCPRs, (b) bauxite.

**
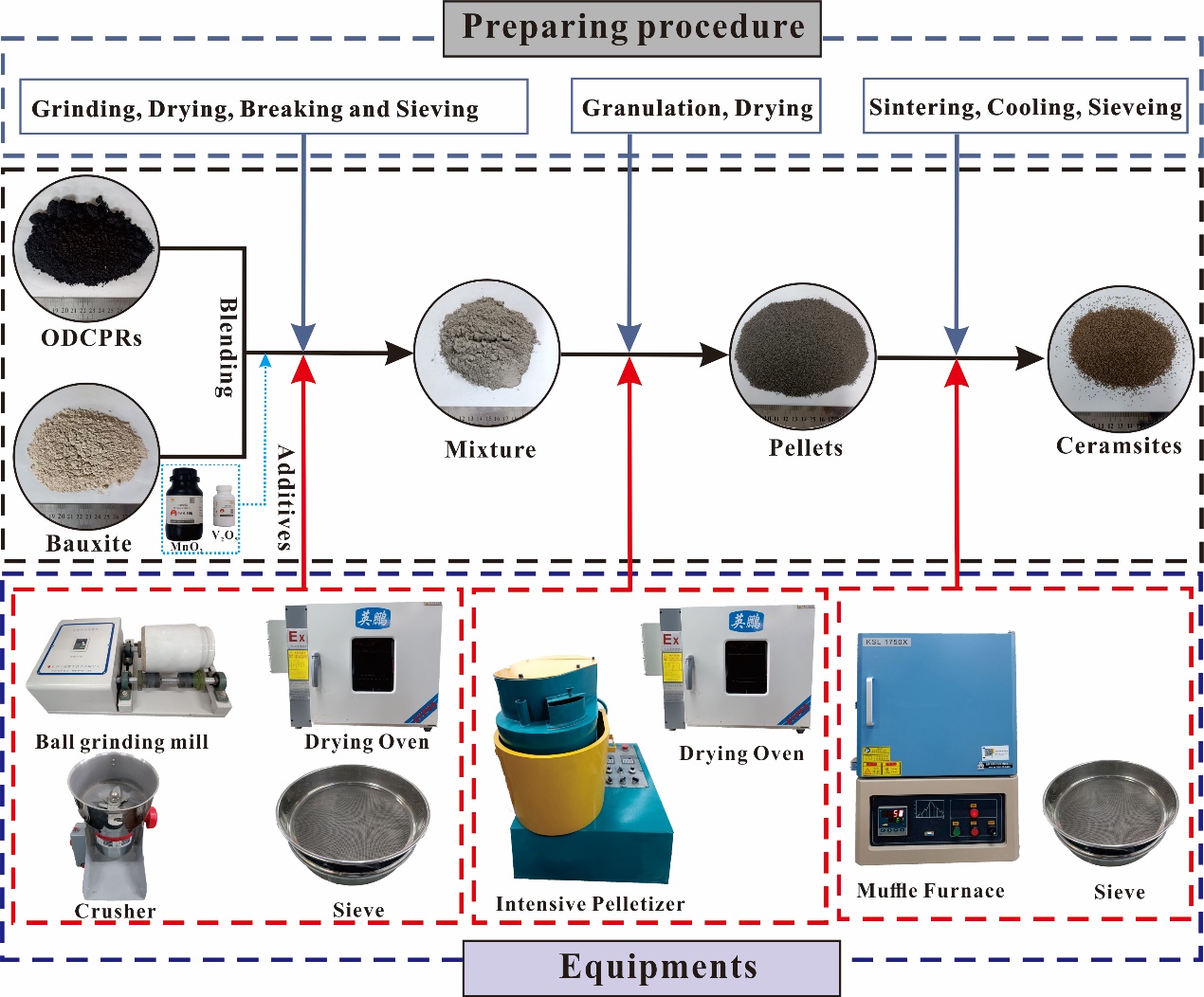
**

**Fig. S2.** Preparation procedure of ceramic proppants.


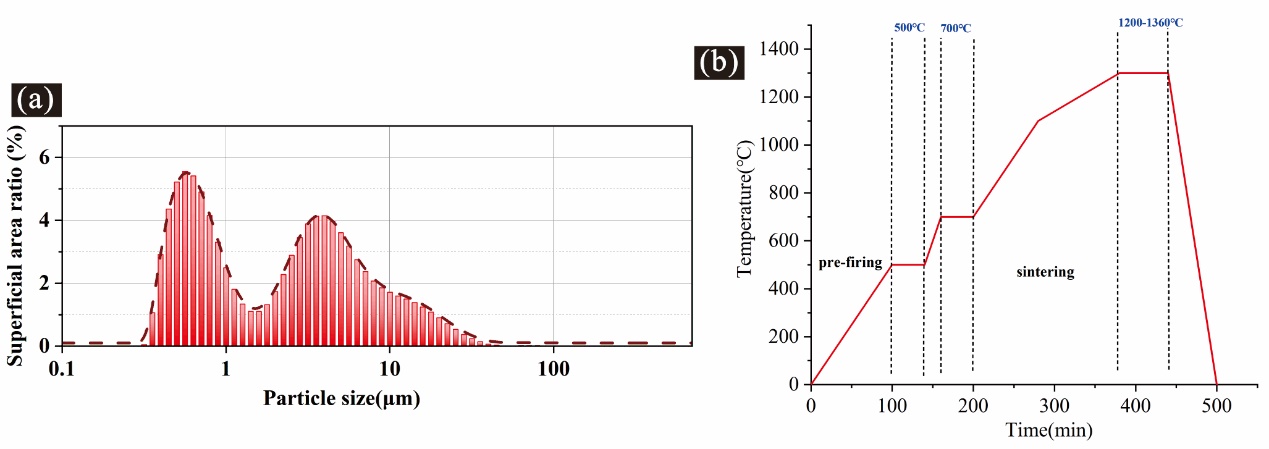


**Fig. S3.** (a) The particle size distribution of the grounded raw material, (b) the sintering systems of ceramic proppants with ODCPRs.


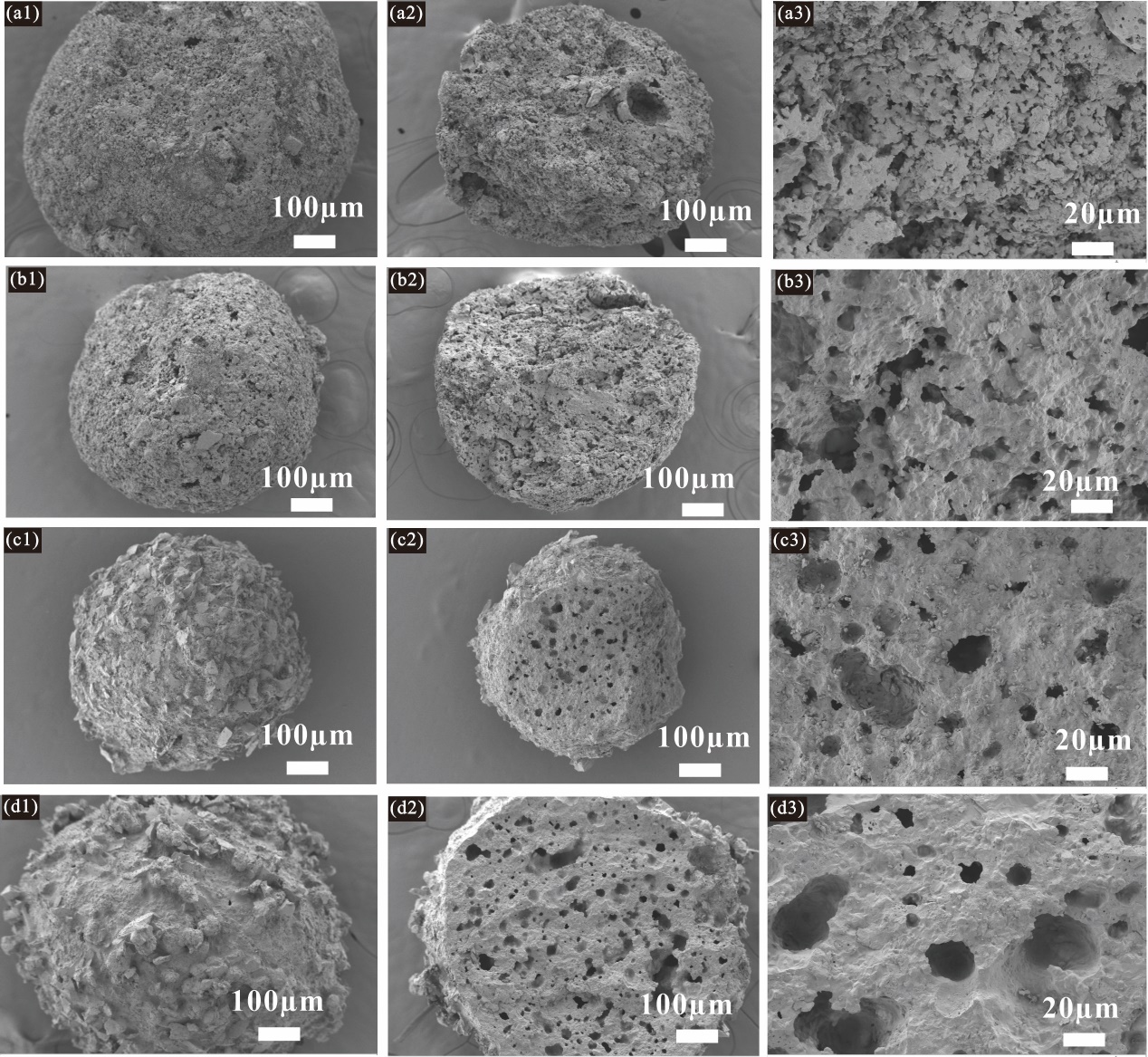


**Fig. S4.** Microscopic diagrams of the ceramic proppant before the acid treatment of the formula B0 at different sintering temperatures: (a) 1240℃; (b) 1280℃; (c) 1320℃; (d) 1360℃.


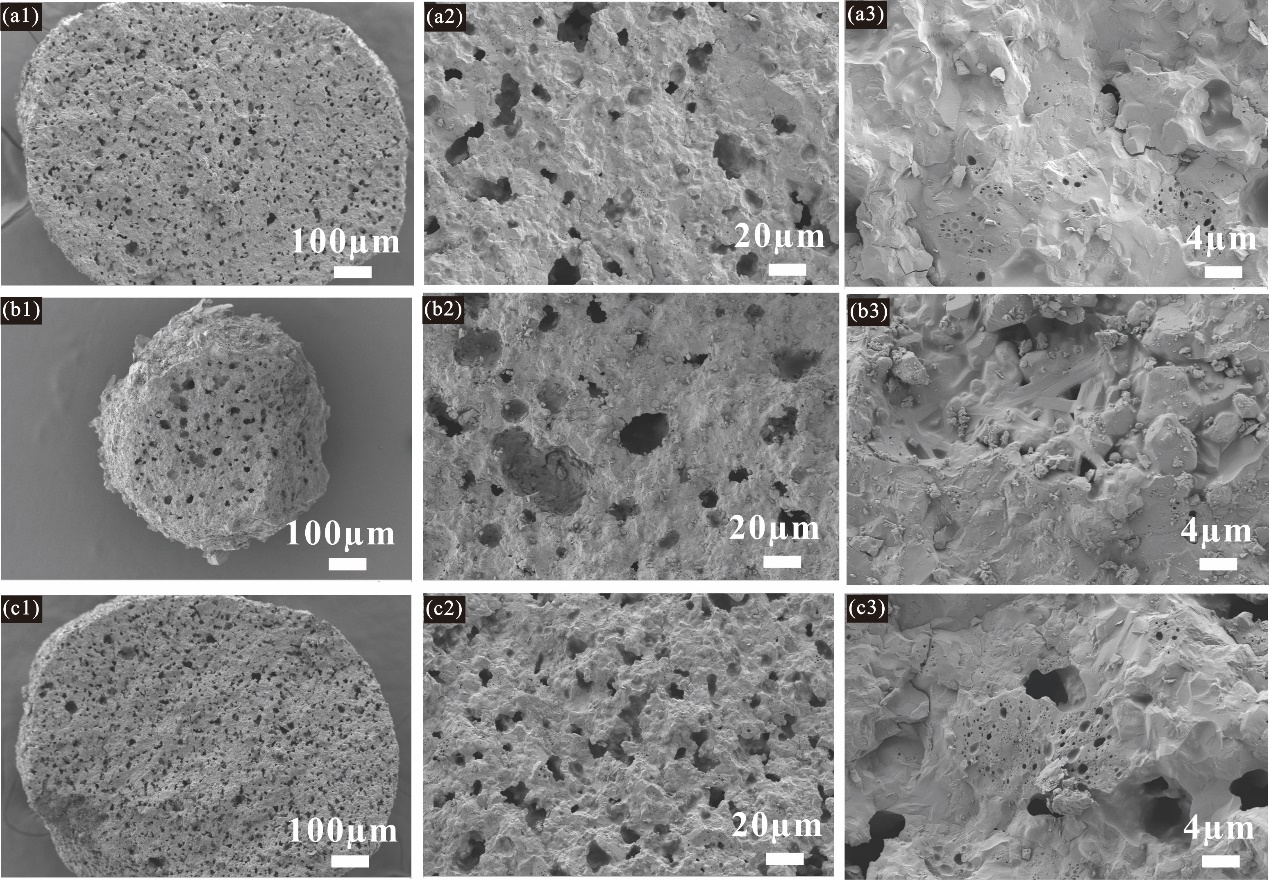


**Fig. S5.** Microscopic diagram of the ceramic proppants before the acid treatment with different ODCPRs contents at their optimum sintering temperature: (a) A0 at 1320℃; (b) B0 at 1320℃, (c) C0 at 1280℃.


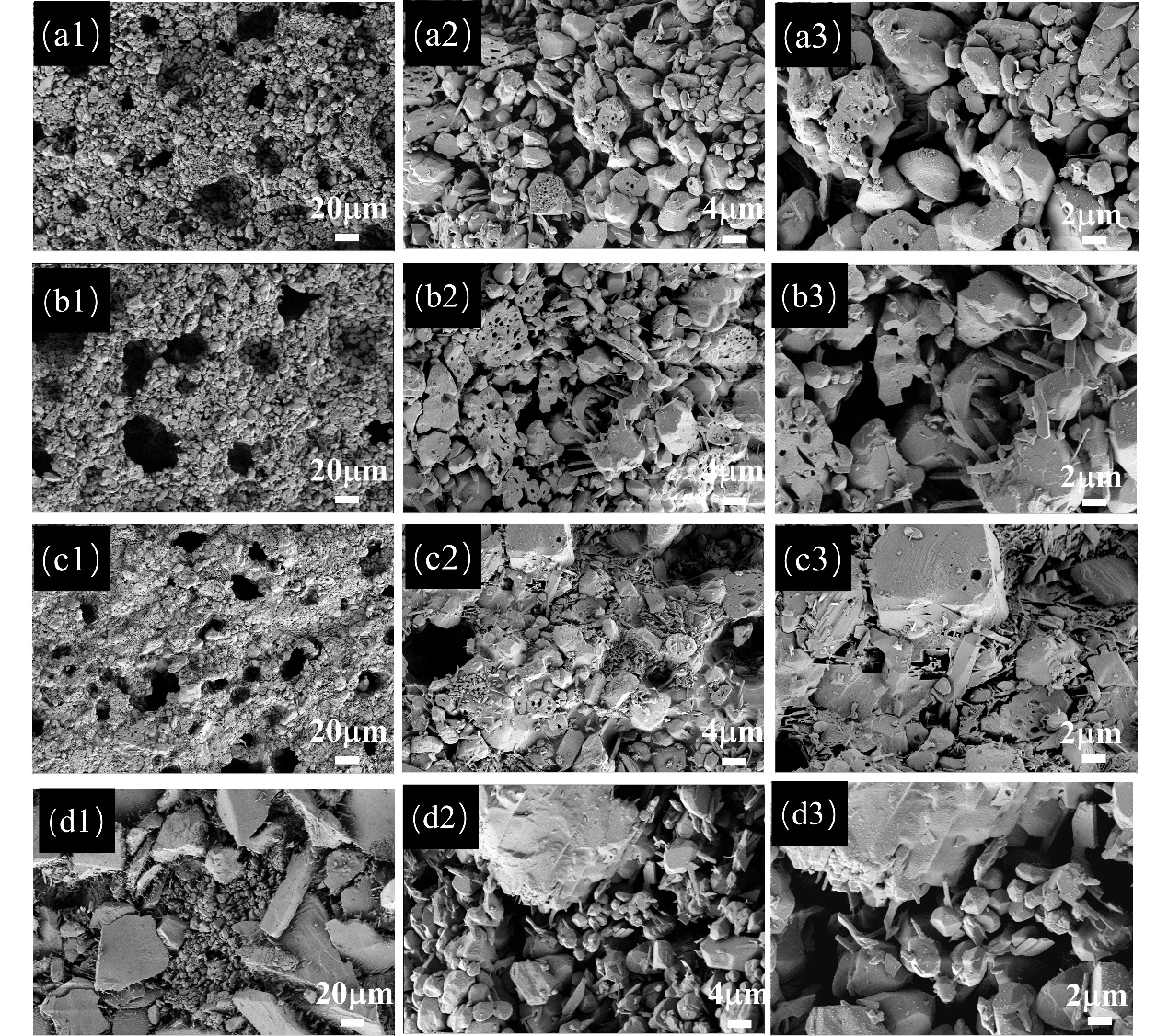


**Fig. S6.** Microscopic diagram of the ceramic proppant before the acid treatment of the formula B0 sintered at 1320℃ with different holding times: (a) 30min; (b) 60min, (c) 90min; (d) 120min.


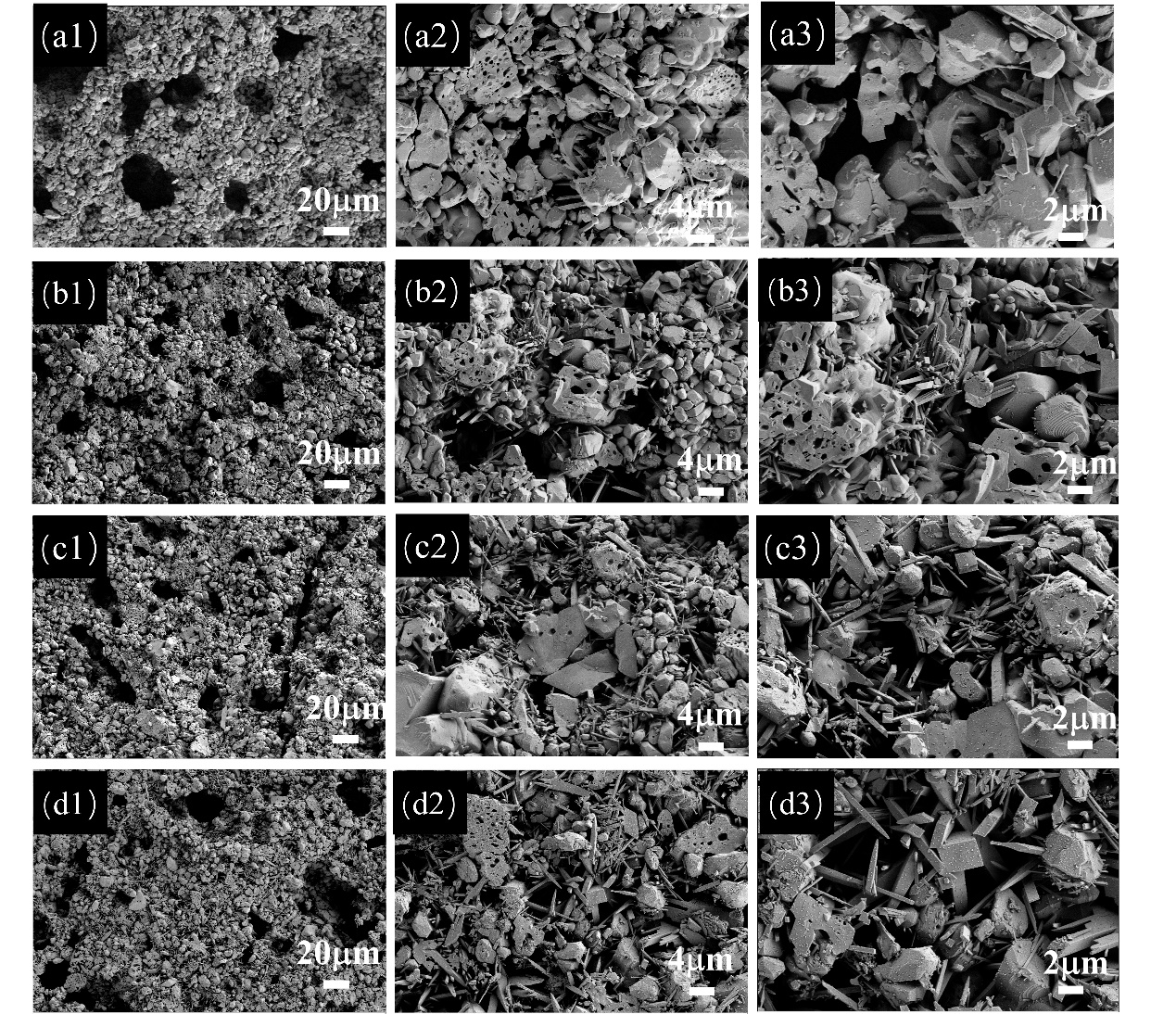


**Fig S7.** Microscopic diagram of the ceramic proppant with different V_2_O_5_ additives after the acid treatment sintered at 1280℃: (a) B0 (b)BV1; (c) BV2; (c) BV3.


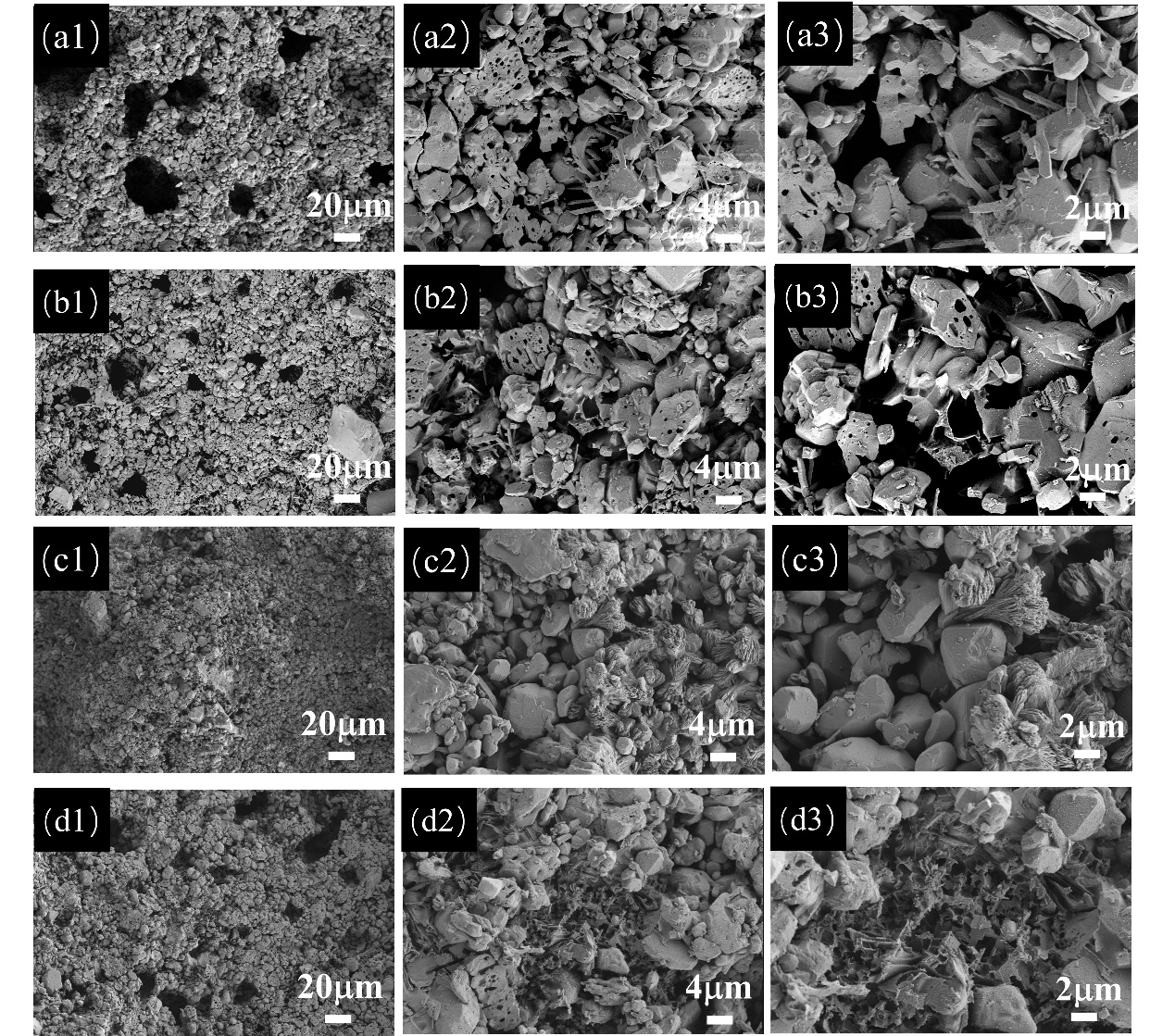


**Fig S8.** Microscopic diagram of the ceramic proppant with different MnO_2_ additives after the acid treatment sintered at 1280℃: (a) BM1; (b) BM2; (c) BM3.

**Table S1.** The chemical composition of the raw materials.

| Components (wt%) | SiO_2_ | Al_2_O_3_ | CaO | TiO_2_ | MgO | Fe_2_O_3_ | SO_3_ | MnO_2_ | Na_2_O | K_2_O | BaO | Cl | P_2_O_5_ | LOI |
| --- | --- | --- | --- | --- | --- | --- | --- | --- | --- | --- | --- | --- | --- | --- |
| ODCPRs | 30.5 | 6.368 | 13.703 | 0.224 | 3.154 | 2.178 | 6.147 | 0.075 | 0.544 | 1.219 | 13.165 | 1.006 | 0.091 | 21.493 |
| Bauxite | 6.16 | 85.91 | 0.34 | 3.89 | 0.48 | 1.53 | 0.14 | / | 0.18 | 0.42 | 0.04 | / | / | 0.26 |
